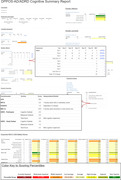# Integrating NACC UDSv3 into non AD/ADRD Cohorts: The Diabetes Prevention Program Outcomes Study in Alzheimer’s Diseases and Related Dementias (DPPOS)‐AD/ADRD Project Experience

**DOI:** 10.1002/alz.086307

**Published:** 2025-01-09

**Authors:** Lindsay Doherty, Jose A. Luchsinger, Marinella Temprosa, Neelesh K. Nadkarni, Terry E. Goldberg, Hanna Sherif, Anna Bowers, Dianilka Martinez, Gerardo J Febres, Danurys Sanchez, James M. Noble, Diabetes Prevention Program Research Group

**Affiliations:** ^1^ George Washington University, Washington, DC USA; ^2^ Columbia University Medical Center, New York, NY USA; ^3^ University of Pittsburgh, Pittsburgh, PA USA; ^4^ Columbia University Irving Medical Center, New York, NY USA

## Abstract

**Background:**

The Diabetes Prevention Program (DPP) was a randomized trial for prevention of diabetes in adults with prediabetes (PreD). The DPP Outcomes Study (DPPOS) is the long‐term epidemiological follow‐up of this cohort, studied continuously for over 25 years. DPPOS Phase 4 (DPPOS‐AD/ADRD) began in 2022, with a primary focus on the nature and determinants of cognitive impairment in the surviving cohort.

In coordination with the National Alzheimer’s Coordinating Center (NACC), DPPOS‐AD/ADRD is implementing their Uniform Data Set version 3 (UDSv3), the gold standard used by Alzheimer’s Disease Research Centers (ADRCs), into DPPOS‐AD/ADRD data collection. Additional cognitive exams and screenings administered as part of the DPPOS for the past 14 years are also continuing in DPPOS‐AD/ADRD. As part of the collaboration, DPPOS‐AD/ADRD will send all UDSv3 data to NACC to be compiled with data from other AD/ADRD‐related studies.

We aimed to merge the UDSv3 elements into the existing longitudinal DPPOS data collection schema, while maintaining full fidelity to the UDSv3, and generate reports for streamlined outcomes adjudication.

**Methods:**

Items from the 16 UDSv3 data forms were compared with those from the forms already collected within DPPOS to harmonize similar items, add UDSv3 items not already collected, and ultimately create a dataset uniform with UDSv3. Forms were adapted for electronic data capture (EDC) using the already‐established and validated platform used for DPPOS data collection.

Automated reports were developed, compiling both current and retrospective neuropsychological scores, to facilitate streamlined adjudication of cognitive outcomes.

**Results:**

Items from UDSv3 were either harmonized with or added to existing DPPOS forms or used for DPPOS‐AD/ADRD in their original form from UDSv3.

Reports for cognitive adjudication display both current and retrospective cognitive data (Figure 1). These reports show scoring trends for repeated assessments over time, adjusted scoring percentiles which are color‐coded according to performance, and interpretations of questionnaire and screening assessment scores. Of approximately 3000 total cognitive adjudications expected, 81 have been initiated and are underway.

**Conclusions:**

The DPPOS‐AD/ADRD project has demonstrated that the UDSv3 can be integrated into established, long‐term studies. The DPPOS‐AD/ADRD project can share best practices for other cohorts seeking to add the NACC‐UDSv3 forms.